# Multilevel Determinants of HIV Prevention Among Urban Refugee Youth in Uganda: Baseline Findings from the Tushirikiane-4-Uthabiti Trial

**DOI:** 10.1007/s10461-025-04941-2

**Published:** 2026-01-05

**Authors:** Moses Okumu, Carmen H. Logie, Zerihun Admassu, Frannie MacKenzie, Lauren S. Tailor, Robert Hakiza, Brenda Katisi, Daniel Kibuuka Musoke, Aidah Nakitende, Catherine N. Nafula, Morris D. C. Komakech, David Okimait, Paul Bukuluki, Peter Kyambadde, Lawrence Mbuagbaw, Liliane C. Windsor

**Affiliations:** 1 School of Social Work, University of Illinois, Urbana-Champaign, 1010 W Nevada St, Urbana, IL 61801 USA; 2 School of Social Sciences, Uganda Christian University, Mukono, Uganda; 3 Factor-Inwentash Faculty of Social Work, University of Toronto, Toronto, ON Canada; 4 United Nations University Institute for Water, Environment, and Health, Hamilton, ON Canada; 5 Centre for Gender & Sexual Health Equity, Vancouver, BC Canada; 6 Women’s College Research Institute, Women’s College Hospital, Toronto, ON Canada; 7 Dalla Lana School of Public Health, University of Toronto, Toronto, ON Canada; 8 The Hospital for Sick Children, Child Health Evaluative Sciences, Toronto, ON Canada; 9 Young African Refugees for Integral Development (YARID), Kampala, Uganda; 10 International Research Consortium (IRC-Kampala), Kampala, Uganda; 11 The Association of Volunteers in International Service (AVSI) Foundation, Kampala, Uganda; 12 School of Nursing, Nipissing University, North Bay, ON Canada; 13 Department of Early Childhood and Pre-Primary Education, Kyambogo University, Kampala, Uganda; 14 College of Humanities and Social Sciences, Makerere University, Kampala, Uganda; 15 Most At Risk Population Initiative Clinic, Mulago Hospital, Kampala, Uganda; 16 National AIDS Control Program, Ministry of Health, Kampala, Uganda; 17 Department of Health Research Methods, Evidence and Impact, McMaster University, Hamilton, Canada; 18 Biostatistics Unit, Father Sean O’Sullivan Research Centre, St Joseph’s Healthcare, Hamilton, ON Canada; 19 Centre for Development of Best Practices in Health (CDBPH), Yaoundé Central Hospital, Yaoundé, Cameroon; 20 Division of Epidemiology and Biostatistics, Department of Global Health, Stellenbosch University, Cape Town, South Africa; 21 Jack, Joseph, and Morton Mandel School of Applied Social Sciences, Case Western Reserve University, 11235 Bellflower Road, Cleveland, OH 44106 USA

**Keywords:** Multilevel intervention, HIV prevention, HIV self-testing, Condom use, Condom self-efficacy, Urban refugees youth, East and Southern Africa, intervención multinivel, prevención del VIH, autoprueba del VIH, uso del preservativo, autoeficacia del preservativo, jóvenes refugiados urbanos, África Oriental y Meridional

## Abstract

While urban refugee youth face HIV vulnerabilities spanning socio-ecological levels, knowledge gaps persist in HIV prevention outcomes. We conducted a baseline analysis of a cohort enrolled in Tushirikiane-4-Uthabiti, an intervention focused on HIV testing practices among urban refugee youth aged 16–24 in Kampala, Uganda (N = 330). Using regression models, we examined the societal, community, and interpersonal factors associated with condom use self-efficacy [CUSE], consistent condom use, HIV self-testing [HIVST] kit access, and recent HIV testing. Most participants were women (53.3%), with a mean age of 21.3 years (SD = 2.9). One-fifth reported consistent condom use (19.1%), over half accessed HIVST kits (56.4%), and recent HIV testing (50.6%). Statistically significant results showed that higher education (β = 0.18, p < 0.001) and financial resilience (β = 0.18, *p* < 0.001) were positively associated with CUSE, whereas food insecurity (β =  − 0.21, *p* < 0.05) was negatively associated with CUSE. Being in a casual relationship (adjusted odds ratio [aOR] 3.33, *p* < 0.05) and CUSE (aOR 1.12, *p* < 0.010) were associated with increased odds of consistent condom use. Higher education (aOR 2.45, *p* < 0.001), adolescent sexual and reproductive health stigma (aOR 1.16, *p* < 0.010), and perceived HIV stigma (aOR 1.05, *p* < 0.05) were associated with increased odds of recent HIVST kit access. Financial resilience (aOR 1.05, *p* < 0.010) and young parenthood (aOR = 2.32, *p* < 0.010) were associated with increased odds of recent last year HIV testing. The findings demonstrate suboptimal HIV prevention outcomes and highlight the need for tailored multilevel interventions to improve the sexual health of urban refugee youth.

## Resumen

Mientras que la juventud refugiada urbana enfrenta vulnerabilidades al VIH en varios niveles socioecológicos, persisten lagunas en el conocimiento sobre los resultados en la prevención del VIH. Realizamos un análisis basal de una cohorte inscrita en Tushirikiane-4-Uthabiti, una intervención centrada en las prácticas de pruebas de VIH entre jóvenes refugiados urbanos de 16 a 24 años en Kampala, Uganda (N = 330). Utilizando modelos de regresión, examinamos los factores sociales, comunitarios e interpersonales asociados con la autoeficacia para el uso del condón [CUSE], el uso consistente del condón, el acceso a kits de autoprueba de VIH [HIVST] y la realización reciente de pruebas de VIH. La mayoría de los participantes eran mujeres (53,3%), con una edad media de 21,3 años (DE = 2,9). Una quinta parte informó uso consistente del condón (19,1%), más de la mitad accedió a kits de autoprueba de VIH (56,4%) y la mitad informó pruebas de VIH recientes (50,6%). Los resultados estadísticamente significativos mostraron que mayor nivel educativo (β = 0,18, p < 0,001) y resiliencia financiera (β = 0,18, p < 0,001) se asociaron positivamente con la autoeficacia para el uso del condón, mientras que la inseguridad alimentaria (β = − 0,21, p < 0,05) se asoció negativamente. Estar en una relación casual (razón de momios ajustada [aOR] 3,33, p < 0,05) y la autoeficacia para el uso del condón (aOR 1,12, p < 0,010) se asociaron con mayores probabilidades de uso consistente de condón. Mayor nivel educativo (aOR 2,45, p < 0,001), el estigma hacia la salud sexual y reproductiva en adolescentes (aOR 1,16, p < 0,010) y el estigma percibido hacia el VIH (aOR 1,05, p < 0,05) se asociaron con mayores probabilidades de acceder recientemente a kits de autoprueba de VIH. La resiliencia financiera (aOR 1,05, p<0,010) y la paternidad/maternidad joven (aOR = 2,32, p < 0,010) se asociaron con mayores probabilidades de haberse realizado una prueba de VIH el último año. Los hallazgos demuestran resultados subóptimos en la prevención del VIH y resaltan la necesidad de intervenciones adaptadas y multinivel para mejorar la salud sexual de la juventud refugiada urbana.

## Introduction

Urban refugee youth in Eastern and Southern Africa (ESA), where more than half of the world’s HIV-affected youth reside [1] and a growing population of refugees live in cities [2–4], face increasing inequities in HIV prevention [5]. In September 2025, Uganda’s refugee population reached nearly 2 million, with a quarter aged 15–24 years, and about 157,000 settled in Kampala [6], a city where the HIV prevalence rate of 11% is more than double the national average [7]. Urban centers such as Kampala offer refugees improved access to education and healthcare but also expose them to heightened HIV risk, driven by pervasive violence, increased sexually transmitted infections (STI) within informal settlements, escalating substance misuse, widespread sex work, and entrenched stigma [8–11]. Systematic reviews [5, 12, 13] show that urban refugee youth are frequently excluded from sexual and reproductive health programs, leaving major gaps in the response to their specific HIV vulnerabilities and needs. To mitigate susceptibility to HIV acquisition among urban refugee youth, contextualized evidence-based HIV prevention interventions are urgently needed.

Guided by the socio-ecological model, multilevel HIV prevention interventions that concurrently address barriers at the societal (e.g., economic insecurity), community (e.g., stigma), and interpersonal (e.g., early parenthood) levels can improve condom and HIV testing practices among youth in humanitarian settings [12, 14, 15]. At the *societal level*, these interventions could address widespread economic challenges in informal settlements that may prevent refugees from affording transportation to clinics to access HIV testing, HIV self-testing [HIVST] kits, or condoms [5, 16, 17]. For instance, employment is a critical economic determinant of HIV prevention uptake, and multiple studies [18, 19] have found a positive association between employment status and HIV testing. Related infrastructural issues may further exacerbate HIV-related risks. For example, a study [20] analyzing population-based data from 112,955 survey participants aged 15–59 years from Zambia, Eswatini, Lesotho, Uganda, Tanzania, and Namibia found that food insecurity was associated with both transactional and condomless sex. Similarly, a previous mixed-methods study [21] of 340 urban refugee youth in Kampala found that both food and water insecurity were associated with reduced sexual and reproductive health access (including HIV testing), and that water insecurity was linked to lower condom use self-efficacy. Education and literacy also play crucial roles in HIV prevention, with studies finding that individuals with higher education levels tend to engage more with HIV services [19]. In contrast, individuals with lower education levels exhibited lower odds of consistent condom use [22] and HIV testing compared to their higher-education counterparts [23]. Indeed, access to higher education may increase young people’s confidence and ability to make decisions regarding how and when to use condoms or test for HIV, and if using HIVST kits, their ability to correctly interpret results [24–26]. Among refugee youth specifically, other findings indicate that poor literacy acts as a barrier to engaging with HIV testing and prevention services, further emphasizing the downstream effect of robust educational infrastructure on condom and HIV testing practices [19]. The absence of tailored HIV prevention programs for urban refugee youth compounds the adverse effects of societal barriers, intensifying their vulnerability to HIV in the context of ongoing displacement and precarious living conditions.

C*ommunity-level* factors, particularly stigma and discrimination, exert a profound influence on condom and HIV testing practices among youth living in humanitarian contexts [10, 11]. For instance, increased adolescent sexual and reproductive health (A-SRH)-related stigma (i.e., devaluing social, cultural, and religious norms and practices toward sexually active youth, youth seeking SRH services, and pregnant and parenting youth) is associated with lower condom use self-efficacy and inconsistent condom use [11], as well as lower HIV testing rates [10]. A recent study [11] focused on urban refugees in Uganda found that intersecting HIV-related and A-SRH stigmas were associated with reduced condom use self-efficacy and actual condom use. Additionally, multiple forms of intersecting stigmas faced by urban refugee youth (i.e., HIV-related stigma, refugee stigma, A-SRH stigma, and sex work stigma) constitute barriers to HIV testing [27]. A systematic review [28] of 10 studies enrolling 3,788 participants found that increased HIV-related stigma decreased the likelihood of HIV testing and care engagement. Therefore, to ensure access and effective utilization of HIV prevention strategies among urban refugee youth, there is a need for evidence to inform the development of community-level stigma-reduction interventions.

At the *interpersonal level*, growing evidence [29, 30] indicates that greater social support is positively associated with increased HIV prevention practices among youth in humanitarian contexts. For example, involving family members and peers, especially those who serve as trusted peer navigators, is linked to a higher incidence of HIV testing [29]. Power dynamics within relationships also influence condom use self-efficacy and HIV testing practices. For instance, partner communication about HIV testing is associated with an increased likelihood of testing [31]. However, there are limited quantitative studies on how relationship dynamics influence HIV prevention outcomes among urban refugee youth. Such relationship dynamics should account for parenthood or caregiving, as the presence of children within a home is also associated with improved HIV testing and prevention practices [19, 32]. For example, one study [33] suggested that childcare responsibilities may motivate parents to seek HIV testing and care, while other research indicates that parenthood can act as a barrier to accessing HIV services, particularly for single parents who may face additional financial or resource insecurities. Scant evidence exists on how young parenthood influences HIV prevention among urban refugee youth, which is a key gap in tailoring interventions for this population.

Given the complexity of the obstacles experienced by urban refugee youth across all levels of the socio-ecological framework in Kampala and beyond, there is a critical need for novel interventions to overcome persistent barriers to HIV prevention and diagnosis. HIVST, recommended by the World Health Organization [34] for adolescents, offers a confidential, accessible, and youth-friendly approach that directly addresses challenges related to stigma, privacy, and service accessibility. This approach enables individuals to privately collect and test their own specimens, such as blood or oral fluid, and to interpret the results themselves. HIVST holds substantial promise for improving HIV status awareness and the uptake of prevention services among urban refugee youth [34]. Encouragingly, a global review [35] found that HIVST implementation not only doubled HIV testing uptake compared to facility-based testing but also increased service providers’ capacity to reach priority populations, including young people aged 16–24 years. In a recent pragmatic controlled trial [26] examining the effectiveness of HIVST with digital support in increasing HIV testing uptake and HIV status knowledge among refugee youth aged 16–24 in Kampala, Uganda. The researchers found that HIVST was highly acceptable among urban refugee youth, with intervention groups exhibiting an uptake rate exceeding 90%. In another study [36] evaluating the effectiveness of combining accessing HIVST kits with 'edutainment' comics among rural settlement-based refugees, findings showed that the intervention increased HIV testing and knowledge of HIV status among refugee youth aged 16–24. Taken together, this emerging body of evidence indicates that HIVST can be effectively implemented in urban and rural refugee contexts. However, there is a need to address multilevel factors that may contribute to the effectiveness and scalability of HIVST interventions among vulnerable youth populations, such as urban refugee youth in low- and middle-income countries, such as Uganda.

To contribute to the evidence-base, this study reports on the baseline HIV prevention outcome data from the Tushirikiane-4-Uthabiti (*translation*: Supporting Each Other For Resilience) trial, a multilevel intervention, described elsewhere [37]. The intervention was co-designed with and for urban refugee youth in Kampala, Uganda. In this study, we describe the participants' sociodemographic characteristics and assess the societal, community, and interpersonal factors associated with HIV prevention outcomes (i.e., condom use self-efficacy, condom use, HIVST kit access, and HIV testing) among urban refugee youth (aged 16–24 years) enrolled in the study. The findings will enable us to 1) gather insights into HIV prevention strategies and vulnerability in a cohort of urban refugee youth in Kampala, Uganda, and 2) assess the potential of addressing multilevel factors (interpersonal, community, and societal levels) to reduce barriers and increase access to and uptake of HIV prevention services.

## Methods

### Study Design and Setting

We analyzed baseline cohort data of urban refugee youth aged 16–24 collected between December 2023 and February 2024, from the ongoing three-arm Tushirikiane-4-Uthabiti trial. Further details regarding the interventions and study design are described elsewhere [37]. The study was conducted in five informal settlements in Kampala, grouped into three clusters based on geographic proximity: 1) Kabalagala and Kansanga, 2) Katwe and Nsambya, and 3) Rubaga. The settlements were chosen based on specific criteria: hosting a large population of urban refugees or displaced persons, similarity in socioeconomic status, healthcare access, languages, and living conditions, and a high prevalence of depressive symptoms among urban refugee youth.

### Study Population and Eligibility Criteria

We recruited 330 youth (110 participants per geographic cluster) aged 16–24 for the study. The eligibility criteria included: 1) residing in one of the five selected informal settlements in Kampala at the time of the study; 2) identified as a displaced person, refugee, or having a parent(s) who were a refugee or displaced; 3) aged 16–24 years; 4) owned or had daily access to a mobile phone; and 5) spoke French, English, Kirundi, Kinyarwanda, or Swahili.

### Participant Recruitment

Peer navigators facilitated participant recruitment through phone calls, in-person meetings, and WhatsApp. The peer navigators (12 in total: six young women and six young men) were experienced health and peer educators from the study communities, identified and recruited by community-based collaborators for their respected status and active involvement in their communities. We employed purposive recruitment methods, such as word-of-mouth and venue-based sampling at community events and refugee agencies to recruit participants. Data collection was conducted in collaboration with refugee (Young Africans for Integral Development [YARID]) and government (Uganda AIDS Control Program, Ministry of Health) agencies. The study received ethical approval from the University of Toronto (#37,496) and Mildmay Uganda Research Ethics Committee (#MUREC-2021–41) and was registered with the Uganda National Council for Science & Technology (#SS1021ES). The clinical trial is registered at ClinicalTrials.gov (NCT06270160).

### Data Collection Procedures

All participants provided informed consent before completing the tablet-based survey. Data were collected by research assistants (RA), who self-identified as refugees and were trained by the Ministry of Health in pre- and post-test HIV counselling, using the SurveyCTO app (Dobility) in all study languages via mobile phones or tablets. The research coordinator conducted daily quality checks on the survey responses to identify any missing or inconsistent data. When such issues were identified, the RAs re-contacted the participants to resolve them, ensuring high data quality and completeness. Given the longitudinal nature of the trial [37], data collection was not anonymized or blinded to the research team. However, strict confidentiality protocols were followed: only authorized research personnel accessed identifying information, and all datasets were de-identified prior to analysis. Data were securely stored, and participant confidentiality was maintained throughout the study period [37]. Each participant received an honorarium of UGX 25,000 (approximately USD 8) for completing a 35–45-min survey.

### Measurement of Variables

#### Outcome Variables

HIV prevention outcomes measured in this study included condom use self-efficacy, consistent condom use, HIVST kit access, and recent HIV testing. *Condom use self-efficacy* was assessed using the 8-item Condom Use Self-Efficacy Scale (CUSES), which measures an individual's confidence in their ability to effectively use condoms (Cronbach’s alpha = 0.89) [38, 39]. Participants rated their confidence on a Likert scale ranging from 1 (Strongly Disagree) to 5 (Strongly Agree). After reverse scoring the negatively worded items, the scores were summed to yield a total score ranging from 8 to 40. Higher scores indicate greater confidence in condom use, whereas lower scores suggest lower confidence. *Consistent condom use* was measured using a single item: “In the past 3 months, how often have you or your sex partner used condoms during intercourse?” The response options were all of the time, some of the time, and none of the time. For analysis purposes, the responses were dichotomized into all of the time vs. some of the time and none of the time. *HIVST kits* access was measured using a single question, “Have you ever received an HIV self-test?” with a yes/no response. *Recent HIV testing* was also assessed using a single-item question, “Have you tested for HIV infections the last one year?” with a yes/no response.

#### Exposure Variables

We assessed factors within the *societal context* (educational level, employment status, water insecurity, food insecurity, and financial resilience), *community context* (adolescent sexual and reproductive health (A-SRH) stigma and HIV-related stigma), and *interpersonal context* (relationship status and parenthood).

Societal level factors included *education level* (less than secondary school vs. post-secondary education) and *employment status* (employed vs. unemployed). Water insecurity was measured using the 12-item household water insecurity experiences scale (total score range: 0–36); a score of 12 or more is considered water insecure (Cronbach’s alpha = 0.92 in the current study) [40]. *Food insecurity* was assessed using a shortened version of the 6-item U.S. Household Food Security Survey Module [41], designed to measure food insecurity within households. Each item was scored as "yes = 1" or "no = 0" with affirmative responses indicating some level of food insecurity. The total score ranged from 0 to 6, with higher scores indicating greater food insecurity. A score of 0–1 indicates high or marginal food security, 2–4 represents low food security, and 5–6 signifies very low food security and *financial resilience* [42, 43] was measured using a 14-item scale, where participants responded on a 5-point Likert scale (1 = strongly disagree to 5 = strongly agree). Total scores were calculated after reversing negatively phrased items, with higher scores indicating greater financial resilience (Cronbach's alpha = 0.90).

Community level factors included *adolescent sexual and reproductive health* (A-SRH) stigma measured using Logie et al.’s 7-item ‘Sexual Activity and Pregnancy Stigma’ subscale, previously validated with urban refugee youth in Uganda [10] (Cronbach's alpha = 0.85). *HIV-related stigma* was assessed using Steward et al.’s 10-item perceived stigma subscale (Cronbach's alpha = 0.90), where higher scores indicated greater levels of HIV-related stigma [44]. Interpersonal level factors included *relationship status* (categorical: no relationship, dating one partner/married, and casual dating/multiple partners), parenthood, where participants were asked if they had children (yes = 1 or no = 0), and *sexual relationship power* was measured using the 15-item Relationship Control subscale of the Sexual and Relationship Power Scale (SRPS) (Cronbach's alpha = 0.90) [45]. Participants responded on a 4-point Likert scale (1 = strongly agree, 4 = strongly disagree). Total scores were calculated, with higher scores indicating greater sexual relationship power.

We assessed the influence of several potential covariates identified in the pre-existing literature. We considered the impact of *sociodemographic characteristics* such as age, gender, place of birth, and duration of residence in Uganda.

### Data Analysis

After data collection was completed, the dataset was systematically coded, cleaned, and analyzed using Stata version 18.0 (StataCorp, College Station, TX). Descriptive statistics were used to summarize the characteristics of the youth participants and the study outcomes across the three geographic locations. For continuous data, means and standard deviations (SD) were calculated, while categorical data were summarized using frequencies and percentages, with chi-square (χ2) tests applied to assess differences between the groups. Logistic and linear regression analyses were conducted to examine the associations between independent and outcome variables. Unadjusted odds ratios (ORs) and unstandardized beta coefficients (*b*) were provided for bivariate analyses, while adjusted odds ratios (aORs) and adjusted standardized beta coefficients (β), along with 95% confidence intervals (CIs), were reported for multivariable analyses. Age, gender, and geographic location were included as confounders in the final multivariable models. All statistical tests were two-tailed, with a significance level of 5%. The goodness of fit for the final model were assessed using the Hosmer–Lemeshow test for the logistic regression model and the adjusted R-squared value for the linear regression model. The model's fit and parsimony were carefully evaluated to maintain an optimal balance between complexity and explanatory power.

## Results

### Sample Characteristics

A total of 330 participants were enrolled in the Tushirikiane-4-Uthabiti trial, consisting of 53.3% women (*n* = 176) with a mean age of 21.3 years (SD = 2.9). Most participants were from the Democratic Republic of Congo (*n* = 257, 77.9%) and had lived in Uganda for six or more years (*n* = 250, 76.4%). Regarding education, approximately three-fifths (*n* = 205, 62.1%) had post-secondary education, and roughly two-thirds of the participants were unemployed (*n* = 227, 69.8%) (Table 1).

**Table 1:** Baseline demographic characteristics of urban refugee youth living in the slums of Kampala, Uganda stratified by geographic location (N = 330)

Participants’ Characteristics	Full sample (*N* = 330)	Kansanga/Kabalagala (*n* = 110)	Rubaga (*n* = 110)	Nsambya/Katwe (*n* = 110)
	*N (%)/ Mean (SD)*	*n* (%)/ Mean (*SD*)	*n* (%)/ Mean (*SD*)	*n* (%)/ Mean (*SD*)
**Socio-demographic variables**				
Age (missing: n = 2)	21.3 (2.9)	21.1(2.6)	21.6(3.1)	21.1(3.0)
Gender				
*Women*	176(53.3%)	58(52.7%)	64(58.2%)	54(49.1%)
*Men*	174(46.7%)	52(47.3%)	46 (41.8%)	56(50.9%)
Country of Birth				
*Democratic Republic of Congo*	257 (77.9%)	90 (81.8%)	61 (55.4%)	106 (96.4%)
*Burundi*	31 (9.4%)	0 (0%)	29 (26.4%)	2 (1.8%)
*Others*	42 (12.7%)	20 (18.2%)	20 (18.2%)	2 (1.8%)
Length of time in Uganda				
< *5 years*	78 (23.6%)	28 (25.5%)	24 (21.8%)	26 (23.6%)
*6–10 years*	148 (44.8%)	43 (39.1%)	46 (41.8%)	59 (53.7%)
> *10 years*	104 (31.5%)	39 (35.4%)	40 (36.4%)	25 (22.7%)
**Societal factors**				
Education level				
*Less than secondary*	125 (37.9%)	33(30%)	37(33.6%)	55(50.0%)
*Post-secondary*	205 (62.1%)	77(70%)	73(66.4%)	55(50.0%)
Employment Status (missing: n = 5)				
*Full-time employed*	37 (11.4%)	8 (7.5%)	17 (15.6%)	12 (11.0%)
*Part-time employed*	61 (18.8%)	20 (18.7%)	21 (19.3%)	20 (18.4%)
*Unemployed*	227 (69.8%)	79 (73.8%)	71 (65.1%)	77 (70.6%)
Water insecurity (missing: n = 11)				
*water secure*	139 (43.6%)	35 (33.3%)	66 (61.1%)	38 (35.8%)
*water insecure*	180 (56.4%)	70 (66.7%)	42 (38.9%)	68 (64.2%)
Food security (missing: n = 3)				
*Food secure*	28 (8.6%)	4 (3.7%)	13 (11.8%)	11(10.1%)
*Food insecure*	299 (91.4%)	104 (96.3%)	97 (88.2%)	98 (89.9%)
Financial resilience (missing: n = 13)	38.98 (8.28)	40.0 (7.4)	36.1 (10.3)	40.9 (5.5)
**Community level factors**				
A-SRH Stigma (missing: n = 12)	4.93 (2.28)	4 (2.45)	6.39 (1.15)	4.36 (2.24)
HIV stigma attitude (missing: n = 21)	22.7(6.1)	20.7(6.7)	25.3(5.9)	21.8(4.6)
**Interpersonal level factors**				
Relationship Status				
*No current partner*	127 (38.5%)	39 (35.4%)	49 (44.6%)	39 (35.5%)
*Dating one partner/married*	159 (48.2%)	56 (50.9%)	35 (31.8%)	68 (61.8%)
*Casual dating/multiple partners*	44 (13.3%)	15 (13.6%)	26(23.6%)	3 (2.7%)
Parenthood				
*No*	270 (81.8%)	98(89.1%)	78 (70.9%)	94 (85.4%)
*Yes*	60 (18.2%)	12(10.9%)	32 (29.1%)	16 (14.6%)
Sexual relationship power (SRP) (missing: n = 32)	48.22 (7.85)	48.03(8.28)	47.17(8.24)	49.76 (6.58)
**Study Outcomes**				
Condom use self-efficacy (missing: n = 21)	28.34(6.48)	26.4(7.67)	29.32(6.37)	29.36 (4.42)
Consistent condom use				
*No*				
*Yes*	123 (58.0%)	44 (63.8)	40 (56.3)	39 (54.2)
HIV testing (missing: n = 4)				
*No*	161 (49.4%)	49 (45.8%)	41 (37.3%)	71 (65.1%)
*Yes*	165 (50.6%)	58 (54.21%)	69 (62.7%)	38 (34.9%)
HIV self testing (missing: n = 2)				
*No*	143 (43.6%)	66 (60.0%)	28 (25.4%)	49 (45.4%)
*Yes*	185 (56.4%)	44 (40.0%)	82 (74.6%)	59 (54.6%)

*SD* standard deviation, *n* missing case

### Multilevel Factors Associated with Condom Practices

#### Condom Use Self-efficacy

The mean score of condom use self-efficacy (CUSE) was 28.34 (SD = 6.48) on a scale ranging from 8 to 40, with significant differences observed among geographic locations. Table 2 shows that after adjusting for age, gender, and geographic location, societal factors associated with CUSE: participants with secondary education or higher had significantly higher CUSE than those with less than secondary education (β = 0.18, 95% CI: [0.08, 0.29]; *p* = 0.001). Conversely, low food security (vs. high/marginal food security) was associated with a lower CUSE (β =  − 0.21, 95% CI: [− 0.30, − 0.11]; *p* = 0.039). Greater financial resilience was positively associated with higher CUSE scores (β = 0.18, 95% CI: [0.07, 0.17]; *p* = 0.001).

**Table 2:** Multilevel factors associated with condom use self efficacy among urban refugee youth living the slums of Kampala, Uganda (N = 330)

Variables	Condom self-efficacy			
	Unadjusted *b* coefficient (95% CI)	P-value	Adjusted β coefficient (95% CI)	P-value
***Sociodemographic factors***				
Age	0.19 (0.13, 0.51)	0.001	0.16 (0.05, 0.27)	0.004
Gender (ref Women)				
*Cisgender Men*	0.22 (0.16, 0.27)	< 0.001	0.20 (0.09, 0.30)	< 0.001
Place of Birth (*ref Democratic Republic of Congo)*				
*Burundi*	0.23 (0.20, 0.26)	< 0.001	0.22 (0.12, 0.33)	< 0.001
*Others*	0.14 (0.10, 0.18)	0.013	0.15 (0.04,0.26)	0.006
Length of time in Uganda (Ref. < 5 years)				
*6–10 Yrs*	0.01 (− 0.07, 0.08)	0.938		
> *10 Yrs*	− 0.07 (− 0.14, − 0.01)	0.325		
***Interpersonal factors***				
Relationship Status (ref *No current partner*)				
*Dating one partner/married*	0.10 (0.60, 0.16)	0.116	0.04 (− 0.08, 0.15)	0.561
*Casual dating/multiple partners*	0.19 (0.15, 0.23)	0.002	**0.18 (0.06, 0.291)**	**0.003**
Parenthood (*ref No*)				
*Yes*	0.04 (− 0.01, 0.08)	0.519		
Sexual Relationship Power	0.04 (− 0.86, 0.95)	0.464		
***Community factors***				
SRH Stigma	− 0.01 (− 0.27, 0.25)	0.877		
HIV Stigma	0.07 (− 0.64, 0.77)	0.254		
***Societal factors***				
Education (ref. *Less than secondary*)				
*Post-secondary*	0.16 (0.11, 0.22)	0.004	**0.18 (0.08, 0.29)**	**0.001**
Employment (*ref. Full-time employed*)				
*Part-time employed*	0.12 (0.05, 0.18)	0.162		
*Unemployed*	− 0.13 (− 0.20, − 0.05)	0.123		
Water insecurity (*ref. water secure*)				
*water insecure*	− 0.09 (− 0.15, − 0.04)	0.104		
Food insecurity (ref *High or marginal food security*)				
*Low food security*	− 0.21 (− 0.31, − 0.11)	0.048	** − 0.21 (− 0.30, − 0.11)**	**0.039**
*Very low food security*	− 0.15 (− 0.25, − 0.05)	0.148	− 0.11 (− 0.21, − 0.01)	0.289
Financial resilience	0.19 (0.74, 1.12)	0.001	**0.18 (0.70, 0.17)**	**0.001**

β-beta coefficient, OR-odds ratio; CI, confidence interval; ref, reference group(s); Adjusted standardized beta coefficient- adjusted for geographic location, age and gender

Bold are socio-ecological factors that are significant

#### Consistent Condom Use

Overall, only one-fifth of the study participants (n = 39, 19.1%) reported consistent condom use, with significant differences observed between the geographic locations. As illustrated in Table 3, in multivariable analyses, interpersonal factors, such as youth engaged in casual dating or having multiple partners, had higher odds of consistent condom use (aOR = 3.33, 95% CI: [1.12, 9.90]; *p* = 0.030). Higher CUSE scores were also significantly associated with consistent condom use (aOR = 1.12, 95% CI: [1.02, 1.22]; *p* = 0.011) (Table 3).

**Table 3:** Multilevel factors associated with consistent condom use among refugee and displaced youth participants in Kampala, Uganda (N = 212)

Variables	Consistent condom use			
	Unadjusted Odd ratio (95% CI)	P-value	Adjusted Odd ratio (95% CI)	P-value
***Sociodemographic factors***				
Age	0.90 (0.81,1.00)	0.061	0.89 (0.79, 0.99)	0.040
Gender (*ref Women*)				
*Cisgender Men*	2.15 (1.23, 3.74)	0.007	2.35 (1.32, 4.16)	0.004
Place of Birth (*ref Democratic Republic of Congo*)				
*Burundi*	1.81 (0.61,5.38)	0.284		
*Others*	0.99 (0.45,2.16)	0.975		
Length of time in Uganda (*ref.* < *5 years*)				
*6–10 Yrs*	0.88 (0.44, 1.76)	0.716		
> *10 Yrs*	0.58 (0.27,1.22)	0.148		
***Interpersonal factors***				
Relationship Status (*ref no current partner*)				
*Dating one partner/married*	1.23 (0.64,2.33)	0.533	1.45 (0.74, 2.86)	0.280
*Casual dating/multiple partners*	2.32 (0.94, 5.75)	0.069	2.50 (0.97, 6.47)	0.059
Parenthood (*ref No*)				
*Yes*	0.39(0.21, 0.72)	0.003	0.55 (0.28, 1.09)	0.088
Sexual relationship power (SRP)	1.02 (0.99, 1.06)	0.213		
Condom use self efficacy	1.08 (1.03, 1.14)	0.003	**1.08 (1.02, 1.14)**	**0.005**
***Community factors***				
A-SRH Stigma	1.05 (0.93, 1.18)	0.427		
HIV stigma	1.01 (0.96, 1.05)	0.832		
***Societal factors***				
Education** (***ref Less than secondary***)**				
*Post-secondary*	0.69 (0.39,1.23)	0.212		
Employment (*ref. full-time employed*)				
*Part-time employed*	0.71(0.27, 1.91)	0.503		
*Unemployed*	0.88(0.37, 2.07)	0.765		
Financial resilience	0.99 (0.96, 1.02)	0.657		

OR-odds ratio; CI, confidence interval; ref, reference group(s); Adjusted OR- adjusted for geographic location, age and gender

Bold are socio-ecological factors that are significant

### Multilevel Factors Associated with HIV Testing Practices

#### Access to HIV Self-test Kits

Access to HIV self-testing kits among participants was 56.4% (n = 185), with significant variability across the study locations (p < 0.001). As shown in Table 4, bivariate analyses revealed that societal (i.e., education) and community (i.e., stigma) factors were positively associated with HIVST kits access. In multivariable analyses, we found that among societal factors, having post-secondary education (vs. less than secondary education) was significantly associated with increased odds of accessing HIVST kits (aOR = 2.45, 95% CI: [1.44, 4.17], *p* = 0.001). Community level factors of stigma-related factors also played a significant role: participants experiencing higher A-SRH stigma (aOR = 1.16, 95% CI: [1.03, 1.29], *p* = 0.011) and higher perceived HIV stigma (aOR = 1.05, 95% CI: [1.01, 1.10], *p* = 0.025) were also significantly associated with increased odds of accessing HIVST kits.

**Table 4:** Multilevel factors associated with lifetime HIV self-testing kits access among displaced youth living the slums of Kampala, Uganda (N = 330)

Variables	Lifetime HIV self test kits access			
	Unadjusted Odd ratio (95% CI)	P-value	Adjusted Odd ratio (95% CI)	P-value
***Sociodemographic factors***				
Age	1.38 (1.26, 1.52)	< 0.001	1.40 (1.27, 1.54)	< 0.001
Gender (*ref Women*)				
*Cisgender Men*	1.40 (0.90, 2.17)	0.135	1.18 (0.72, 1.92)	0.506
Place of birth (ref Democratic Republic of Congo)				
*Burundi*	6.80 (2.31, 20.00)	< 0.001	6.09 (1.90, 19.46)	0.002
*Others*	2.84 (1.37, 5.90)	0.005	2.98 (1.33, 6.67)	0.008
Length of time in Uganda (*ref.* < *5 years*)				
*6–10 Yrs*	2.84 (1.61, 5.04)	< 0.001	2.77 (1.46, 5.23)	0.002
> *10 Yrs*	3.04 (1.65, 5.61)	< 0.001	2.98 (1.49, 5.94)	0.002
***Interpersonal factors***				
***Relationship Status*** *(ref no current partner)*				
*Dating one partner/married*	1.48 (0.92, 2.37)	0.105	0.86 (0.50, 1.48)	0.596
*Casual dating/multiple partners*	2.21 (1.07, 4.56)	0.032	1.56 (0.70, 3.49)	0.278
Parenthood (*ref No*)				
*Yes*	2.04 (1.12, 3.72)	0.020	1.25 (0.62, 2.51)	0.534
Sexual relationship power (SRP)	0.99 (0.96, 1.02)	0.729		
Ever had sex (*ref No*)				
*Yes*	2.04 (1.29, 3.20)	0.002	0.77 (0.44, 1.36)	0.367
***Community factors***				
SRH Stigma	1.10 (0.99, 1.21)	0.059	**1.16 (1.03, 1.29)**	**0.011**
HIV stigma	1.06 (1.02, 1.10)	0.003	**1.05 (1.01, 1.10)**	**0.025**
***Societal factors***				
Education (*ref less than secondary*)				
*Post-secondary*	2.52 (1.60, 3.99)	< 0.001	**2.45 (1.44, 4.17)**	**0.001**
Employment (*ref. full-time employed*)				
*Part-time employed*	0.80 (0.32, 1.98)	0.628	0.68 (0.25, 1.84)	0.448
*Unemployed*	0.38 (0.17, 0.82)	0.013	0.62 (0.26, 1.46)	0.270
Water insecurity (*ref. water secure*)				
*Water insecure*	0.64 (0.41,1.01)	0.056	0.65 (0.39, 1.08)	0.098
Food insecurity frequency High or marginal food security				
*Low food security*	1.40 (0.58, 3.34)	0.452		
*Very low food security*	0.46 (0.20, 1.04)	0.062		
Financial resilience	1.01 (0.98, 1.03)	0.606		

OR-odds ratio; CI, confidence interval; Ref, reference group(s); Adjusted OR- adjusted for geographic location, age and gender

Bold are socio-ecological factors that are significant

#### Recent HIV Testing

Only half of the youth (n = 165, 50.6%) reported having received an HIV test in the past year, with significant differences noted across geographic locations. Factors associated with higher odds of recent HIV testing in the bivariate analyses included age, place of birth, having children, sexual history, water insecurity, education level, and employment status (Table 5). In multivariable analyses, interpersonal factors of young parenthood had over twice the odds of reporting a recent HIV test compared to non-parenthood (aOR = 2.32; 95% CI: [1.17, 4.62]; *p* = 0.016). The societal factor of higher financial resilience was significantly associated with increased odds of recent HIV testing (aOR = 1.05; 95% CI: [1.02, 1.08]; *p* = 0.003).

**Table 5:** Multilevel factors associated with recent HIV testing among displaced youth living in the slums of Kampala, Uganda (n = 330)

Variables	Recent HIV Test			
	Unadjusted Odd ratio (95% CI)	P-value	Adjusted Odd ratio (95% CI)	P-value
***Sociodemographic factors***				
Age	1.30 (1.20, 1.42)	< 0.001	1.31 (1.20, 1.43)	< 0.001
Gender (ref Women)	1.00 (0.65, 1.55)	0.988	0.85 (0.52, 1.37)	0.498
*Cisgender Men*				
Place of Birth (*ref Democratic Republic of Congo*)				
*Burundi*	2.56 (1.16, 5.65)	0.020	1.93 (0.83, 4.51)	0.126
*Others*	3.21 (1.54, 6.70)	0.002	2.47 (1.11, 5.49)	0.026
Length of time in Uganda (*ref.* < *5 years*)				
*6–10 Yrs*	1.09 (0.63, 1.90)	0.751	1.04 (0.57, 1.89)	0.901
> *10 Yrs*	1.44 (0.80, 2.61)	0.227	1.17 (0.61, 2.23)	0.638
***Interpersonal factors***				
Relationship Status (*ref no current partner*)				
*Dating one partner/married*	1.38 (0.86, 2.21)	0.181	0.95 (0.56, 1.61)	0.850
*Casual dating/multiple partners*	1.77 (0.88,3.56)	0.106	1.07 (0.50, 2.29)	0.861
Parenthood (*ref no*)				
*Yes*	3.30 (1.77, 6.13)	< 0.001	**2.32 (1.17, 4.62)**	**0.016**
Sexual relationship power (SRP)	1.00 (0.97, 1.03)	0.915	1.00 (0.97,1.03)	0.946
Ever had sex (*ref no*)				
*Yes*	2.80 (1.76, 4.46)	< 0.001	1.65 (0.95, 2.86)	0.073
***Community factors***				
A-SRH Stigma	1.04 (0.95, 1.15)	0.382	1.09 (0.98, 1.22)	0.094
HIV stigma	0.99 (0.96, 1.04)	0.956	0.99 (0.95, 1.03)	0.678
***Societal factors***				
Education (*ref less than secondary*)				
*Post-secondary*	2.06 (1.31,3.26)	0.002	1.47 (0.89,2.43)	0.129
Employment (*ref full-time employed*)				
*Part-time employed*	0.96 (0.40, 2.30)	0.927	0.84 (0.33, 2.13)	0.715
*Unemployed*	0.37 (0.18, 0.78)	0.009	0.53 (0.24, 1.16)	0.112
Water insecurity (*ref water secure*)				
*Water insecure*	0.63 (0.40, 0.98)	0.040	0.63 (0.39, 1.03)	0.066
Food insecurity (ref high or marginal food security)				
*Low food security*	0.63 (0.27,1.45)	0.278	0.55 (0.22, 1.38)	0.206
*Very low food security*	0.83 (0.37, 1.85)	0.646	0.73 (0.30, 1.79)	0.496
Financial resilience	1.04 (1.01, 1.07)	0.009	**1.05 (1.02, 1.08)**	**0.003**

OR-odds ratio; CI, confidence interval; ref, reference group(s); Adjusted OR- adjusted for geographic location, age and gender

Bold are socio-ecological factors that are significant

## Discussion

Guided by the socio-ecological model, this study provides a baseline analysis of HIV prevention practices and multilevel determinants among a cohort of urban refugee youth participating in the Tushirikiane-4-Uthabiti trial in Kampala, Uganda. Our findings show that both past-year HIVST kit access, HIV testing and consistent condom use remain markedly below national [19] and international targets [46], despite participants reporting moderate condom use self-efficacy. These prevention gaps may reflect COVID-19 related disruptions to health services, as well as ongoing barriers such as limited educational opportunities, food insecurity, and intersectional stigma [21, 47–49]. Societal factors, including post-secondary education and financial resilience, were associated with increased condom use self-efficacy and HIVST access, whereas food insecurity hindered these protective behaviors. At the community level, greater A-SRH and higher perceived HIV-related stigma were associated with increased HIVST access. Consistent condom use was influenced by interpersonal dynamics, particularly relationship context and negotiation skills. Taken together, these findings highlight the pressing need for multilevel, contextually tailored interventions that address intersecting vulnerabilities to improve HIV prevention outcomes among urban refugee youth.

Corroborating prior studies, we found that post-secondary education was associated with higher condom use self-efficacy [50, 51] and greater odds of accessing HIVST kits [52], highlighting a key opportunity for societal interventions that increase access to education for urban refugee youth. Lower education levels may be linked to poor literacy rates among refugee youth [19, 53], which could act as a barrier to negotiating condom use or effectively accessing and using HIVST kits. Indeed, multiple studies [50, 51] of ESA adolescents and young adults have identified an association between higher education levels and increased condom use self-efficacy. Further corroborating prior studies [24, 54], our analysis found that refugee youth with higher education levels had greater confidence in their ability to access HIVST kits. Evidence suggests that higher education is associated with increased awareness and access to information about HIV and HIVST, and potentially increased willingness to access and use HIVST kits [55]. Thus, access to higher education may bolster refugee youth’s health literacy skills, enabling them to understand and properly interpret HIVST instructions and results [56]. Prior studies [27, 57] have also shown that low literacy levels represent a key barrier to access and usage of HIVST kits, as individuals with low literacy levels may be susceptible to misinformation or misinterpretation of results. Therefore, improving literacy and, by extension, health literacy among urban refugee youth represents an opportunity to expand sexual health knowledge and skills, which, in turn, will boost their ability to access, negotiate for, and consistently and effectively use condoms and HIVST kits. Such an initiative would not only align with the goals of non-HIV-related initiatives but also leverage existing infrastructure (e.g., schools and other literacy programs) to promote HIV preventive practices among a vulnerable population. Therefore, to maximize HIVST access and uptake among urban refugee youth, new programs should use diverse distribution and knowledge access points, such as pharmacies, shopping centers, clinics/hospitals, schools, bars/clubs, sporting venues, and sexual partners [58, 59]. These programs should employ vernacular languages [26, 37, 57] and graphic medicine (comics) [36, 60, 61] to increase the reach and usage of HIV prevention information by low-literacy displaced youth.

We also observed an association between high financial resilience and increased condom use self-efficacy and likelihood of HIV testing among our urban refugee youth sample, underscoring the critical role of economic stability in shaping sexual health practices and access to healthcare services in humanitarian settings. Economic stability increases refugee youth’s ability to exercise greater control over their sexual decision-making.For instance, a related finding of this study, namely that food insecurity was inversely related to condom use self-efficacy, corroborates prior findings [62], illustrating how, in the context of economic instability, youth may have less ability to negotiate condom use with partners upon whom they may depend economically. Financial resilience may also mitigate barriers (e.g., transportation costs, lost wages) to accessing health services while reducing urban refugee youth’s engagement in high-risk practices (e.g., transactional sex), which are linked to lower rates of HIV testing in this population. Consequently, these results highlight the importance of integrating economic strengthening components into HIV prevention and testing interventions targeting urban refugee youth living in informal settlements.

We found that high community-level A-SRH and perceived HIV-related stigma were associated with a greater likelihood of accessing HIVST kits. This finding reinforces the notion that young people who are more stigmatized may prefer the confidentiality of HIVST, as it can reach priority populations by providing increased privacy, confidentiality, and autonomy [35]. Prior refugee youth research in Uganda [27, 63] have documented that intersecting stigma increases interest in HIVST over clinic-based testing. Furthermore, studies [26, 36, 64] have shown that HIVST is highly acceptable, especially among populations experiencing greater vulnerability due to structural and social factors (e.g., urban refugee youth). In these populations, the use HIVST can increase testing rates by empowering individuals to test themselves privately, and (depending on testing results) can increase linkage to HIV treatment or prevention services. However, some studies [27, 29, 59] have highlighted the need to diversify the distribution of HIVST kits beyond public facilities due to fears of stigma and discrimination in public sites. Thus, to fully realize the stigma-reducing potential of HIVST, distribution strategies leveraging pharmacies, supermarkets, online stores, and youth-friendly testing centers could minimize exposure to stigma and enhance both the acceptability and uptake of HIVST among populations most affected by HIV-related stigma. These cross-sectional findings, though promising, require further longitudinal exploration to better determine whether stigma predicts increased use of HIVST and whether stigma changes following HIVST access and utilization.

Finally, our findings indicate that at the interpersonal level, individuals in committed relationships and those with children were more likely to report consistent condom use and HIV testing than those without children [19, 32]. These associations may be attributed to improved communication, trust, or shared responsibility between partners, which facilitate mutual decision-making concerning sexual health and service utilization [19]. When interpreted through a rights-based framework, healthy sexual practices and access to HIV prevention are regarded as fundamental rights that must be ensured for all young individuals, irrespective of their relationship or parental status. Thus, opportunities for positive interpersonal dynamics are influenced by broader social and structural determinants, including stigma, gender norms, and access to supportive services. These findings underscore the significance of interventions that enhance partner communication and negotiation skills while addressing the intersecting barriers that limit youth agency in diverse relational contexts. Further, we found that condom use self-efficacy is a critical factor in promoting consistent condom use. Strategies to increase condom use self-efficacy should span socio-ecological levels, integrating intrapersonal (e.g., knowledge), interpersonal (e.g., relationship dynamics), and structural (e.g., condom access) factors [39, 65, 66]. These strategies can empower urban refugee youth with the knowledge, relationships, and infrastructural support they need to effectively negotiate safer sex practices [31]. Future HIV prevention interventions must account for the unique social contexts and relationships of urban refugee youth to improve their overall sexual health outcomes.

### Limitations

This study has several strengths, including the large recruitment and enrollment of a cohort of urban refugee youth with diverse HIV prevention needs. Despite this, our findings should be interpreted in light of their limitations. One potential limitation of this baseline analysis is the cross-sectional approach. Although it provided prevalence data, namely condom (i.e., condom self-efficacy and consistent condom use) and HIV (i.e., HIVST access and HIV testing) practices, the observed associations must be interpreted with caution, as they do not imply causal relationships. Second, the absence of data on other key populations, such as sexually diverse individuals, and people who use substances, limits our ability to assess how intersecting identities and forms of stigma that may shape HIV testing practices in these groups. Future research should consider these intersectional dynamics among urban refugee youth in low- and medium-income countries. Third, the outcome measures were self-reported, which may have been affected by social desirability bias. However, self-reported measures have been shown to be reliable for reporting HIVST and HIV testing [67]. To improve the quality of our condom practice assessment, we used validated measures [19, 37, 39]. Furthermore, to limit the impact of social desirability bias on our findings, the survey was contextualized to the setting and administered in a local language selected by the participants. In addition, participants were provided with survey tablets to complete HIV vulnerability-related questions privately, further minimizing concerns regarding the impact of social desirability bias on our study findings.

## Conclusion

The uptake of HIV preventive measures among urban refugee youth living in the slums of Kampala, Uganda during the baseline period was suboptimal. Our findings underscore the need for targeted multilevel HIV prevention strategies for displaced populations, particularly urban refugee youth, to address their unique vulnerabilities and ensure progress towards the UNAIDS goal of 95% of people globally knowing their HIV status by 2030 [46]. Our findings regarding the association between socio-ecological factors and reduced uptake of HIV preventive measures underline the need for multilevel, multicomponent interventions, such as initiatives combining economic strengthening interventions that address structural factors of education, food security, and financial resilience with stigma-reduction strategies that address community factors of HIV-related stigma and A-SRH. At the interpersonal level, there is a need to develop young refugees’ condom negotiation strategies [31, 65, 68] to increase consistent condom use, thereby reducing HIV acquisition and transmission. Future research studies from the Tushirikiane-4-Uthabiti project will provide longitudinal evidence from a cohort of urban refugee youth living in urban slums in Africa's largest refugee-hosting country, Uganda.
